# Ochratoxin A in bulk consignments of wheat, durum wheat, and barley sampled before and after trans-Pacific shipment

**DOI:** 10.1007/s12550-026-00642-4

**Published:** 2026-02-23

**Authors:** Sheryl A. Tittlemier, Atsushi Sekiyama, Yukiko Yamada

**Affiliations:** 1 Grain Research Laboratory, Canadian Grain Commission, Winnipeg, MB Canada; 2 Ministry of Agriculture, Forestry and Fisheries, 1-2-1 Kasumigaseki, Chiyoda-ku, Tokyo, 100-8950 Japan

**Keywords:** OTA, Heterogeneity, Variance, Sampling, Grains, Mycotoxin, Transport

## Abstract

Samples of 165 bulk wheat, durum wheat, and barley consignments were taken during loading in western Canada and unloading in Japan from September 2018 through September 2022. The 330 paired samples were analyzed for ochratoxin A (OTA). OTA was observed at concentrations ranging from 0.16 to 3.3 µg/kg, predominantly in wheat and durum wheat. Concentrations of OTA were not consistently greater in unloading as compared to loading samples from the same bulk consignments, nor was there correlation between OTA concentration at unloading and the duration of transport, suggesting the storage conditions during transport were not conducive to *Penicillium verrucosum* proliferation and OTA production during the mean transport time of 29 days. A lack of correlation of OTA concentrations in loading and unloading samples reflected the heterogeneous distribution of this mycotoxin in bulk grain and emphasized the importance of proper sampling to mitigate the impact of the heterogeneous distribution on variance of OTA measurements, and on inspection of compliance.

## Introduction

Ochratoxin A (OTA) is a mycotoxin produced by some *Aspergillus* and *Penicillium* species of fungi. However, *P. verrucosum* is more relevant in temperate climates and is associated with stored cereal grains (Duarte et al. 2010). The global nature of the grain trade results in large volumes of cereal grains, such as wheat, durum wheat, and barley, being stored and transported long distances potentially under conditions that could support *P. verrucosum* growth and OTA production. Monitoring studies have reported the presence of OTA in cereal grain shipments (De Pace et al. 2012; Tittlemier et al. 2014; Sekiyama et al. 2025), but did not investigate whether OTA production occurred during transport.

The presence of OTA in certain commodities and foods is regulated in some jurisdictions because of the nephrotoxic hazards of this mycotoxin. The Codex Alimentarius Commission adopted a Maximum Level of 5 µg/kg for raw wheat (including common wheat, durum wheat, spelt and emmer), barley and rye (Codex Alimentarius Commission 2023) to be applied in international trade in 2009 (Codex Alimentarius Commission 2008). Therefore, monitoring and compliance testing for OTA often occur as part of trade of these grains.

*P. verrucosum* growth and production of OTA during storage of cereal grains, and not in the field during the growing season, contributes to a very heterogeneous distribution in bulk wheat consignments (Tittlemier et al. 2025), as well as smaller volumes of wheat (Biselli et al. 2008) and other cereal grains (Whitaker et al. 2015). This heterogeneous distribution leads to a particularly challenging situation for sampling (Tittlemier et al. 2011), requiring adherence to Theory of Sampling principles to mitigate impacts of grain consignment heterogeneity on test results variance (Tittlemier and Whitaker 2025).

In this study, targeted surveillance was established to examine OTA occurrence in bulk shipments of wheat loaded on the west coast of Canada, transported across northern Pacific and unloaded in various ports in Japan. This surveillance activity produced a unique sample set with pairs of independent samples representing the grain at loading as well as unloading to investigate whether or not changes in OTA presence occurred during transport.

## Materials and methods

### Samples

**Fig. 1:**
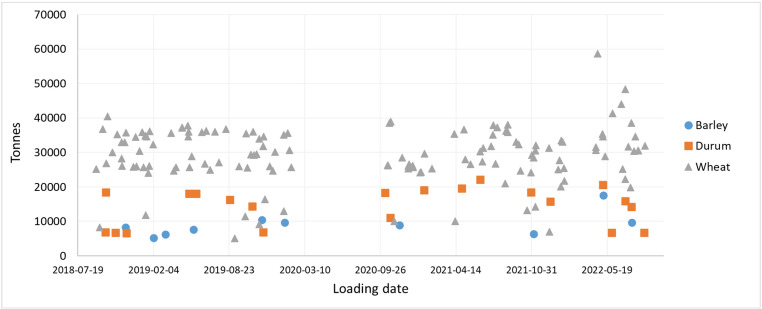
Loading date and size of bulk consignments sampled

Bulk consignments of common wheat (Canada Western Red Spring, CWRS; *n* = 134), durum wheat (Canada Western Amber Durum, CWAD; *n* = 21), and barley (*n* = 10) loaded in Canada for export to Japan were included in the study. The 165 bulk consignments were loaded between September 5, 2018 and August 27, 2022 (Fig. 1). The bulk consignments ranged in size from 5,150 to 17,612 tonnes for barley, 6,592 − 22,200 tonnes for durum, and 5,144 − 58,737 tonnes for wheat. Consignments were sampled by: Canadian Grain Commission grain inspectors during loading in Canada; and by two third-party organizations contracted by the Ministry of Agriculture, Forestry and Fisheries during unloading in Japan. As a result, duplicate independent samples representing each consignment were obtained. There was a period of time between February 4, 2020 and October 7, 2020 in Canada or between March 1, 2020 and October 28, 2020 in Japan, when consignments were not sampled because of restrictions in place due to Covid-19.

### Sampling and OTA analysis at loading

Moving grain was sampled during the loading of vessels for export of the bulk consignments to Japan. Canadian Grain Commission-approved automated cross-cut samplers and dividers were used to obtain increments at a constant interval throughout the entire loading process (Canadian Grain Commission 2015). The increments were combined into an aggregate sample that was representative of the entire bulk consignment. A Boerner divider (Seedburo Equipment Co.) was used to divide the aggregate sample into a 10 kg laboratory sample.

Test portions for OTA analysis were prepared from the 10 kg laboratory sample of whole grain according to the process described in Tittlemier et al. (2012). The 10 kg laboratory sample of whole grain was comminuted using a rotor beater mill (Retsch SR 300 with a 0.75 mm sieve screen) coupled with a vibratory feeder (Retsch DR 100). After comminution, the entire mass of ground grain was divided into 10 × 1 kg portions on a rotary sample divider (Materials Sampling Solutions, Southport, Australia). After the first division into 10 × 1 kg, all 10 portions were recombined in the rotary sample divider hopper and divided into 10 × 1 kg again. A 1 kg portion of ground grain was randomly selected and further divided by rotary sample division into 10 × 100 g. Test portions of 100 g were stored at room temperature in closed high-density polyethylene or polypropylene containers. Samples were analyzed for OTA on average within 11 days of loading.

Test portions were extracted and analyzed for OTA using ultra-high pressure liquid chromatography with fluorescence detection (Tittlemier et al. 2012). Briefly, chloroform and 0.1 mol/L phosphoric acid (6:1, v/v) was shaken with approximately 100 g of ground grain mixed with celite for 15 min and then centrifuged. An aliquot of the filtered organic layer was exchanged into dichloromethane and further purified using a silica gel solid phase extraction cartridge, using toluene-acetic acid (9:1, v/v) to elute OTA. The eluate was evaporated to dryness under N_2_, reconstituted in acetonitrile-0.5% acetic acid (1:1, v/v), filtered, and analysed. Sample extracts were chromatographed on a BEH C 18 column (1.7 μm particle size, 2.1 × 50 mm) using a mobile phase of water adjusted to a pH of 4.0 with acetic acid and acetonitrile in a gradient program. Excitation and emission wavelengths of 340 nm and 465 nm, respectively, were used to analyze OTA. It was positively identified and quantified if its retention time was within 0.1 min of the average retention time in the external calibration standards and the peak had a signal to noise ratio greater than 9:1. Concentrations were recovery corrected using the percent recovery of OTA from a fortified matrix blank analysed with every sample batch. The limit of quantitation (LOQ) of OTA was 1.0 µg/kg, calculated as the concentration of OTA in sample extract required to produce a peak with signal to noise ratio of 9:1. A blank matrix sample fortified with OTA at 5.0 µg/kg and a wheat reference material were included in each analytical run to monitor method performance. Expanded measurement uncertainty that incorporated uncertainty contributions from accuracy and precision was estimated to be 51.5%, consistent with other reported measurement certainty estimations for OTA (Biselli et al. 2008; Andersson et al. 2011).

### Sampling and OTA analysis at unloading

The bulk consignment took approximately 3–5 weeks to cross the Pacific Ocean. Generally, each bulk consignment was unloaded at multiple berths at 3 to 5 different ports in Japan. Ministry of Agriculture, Forestry and Fisheries selected one unloading berth from which to sample the portion of the bulk consignment that was being unloaded at the selected berth as described in Sekiyama et al. (2025). Moving grain was sampled manually using a hand scoop while the grain was transferred from conveyer to silo during the entire unloading process to take 100 incremental samples, each consisting of 100 g, to produce a laboratory sample of 10 kg that represented the portion of the bulk consignment unloaded at one berth in Japan. Approximately 20% of the whole bulk consignment was sampled at unloading, as compared to 100% of the full consignment sampled during loading.

The 10 kg laboratory sample of whole grain was mixed and comminuted using a rotor beater mill (Retsch ZM200 with a 1.0 mm–0.5 mm sieve screen). The comminuted 10 kg of grain was manually sampled by taking 10 × 100 g portions to prepare a 1 kg subsample. The subsamples were manually shaken thoroughly to mix and then stored under frozen conditions in plastic bags. A 25.0 g test portion was taken from the 1 kg subsample by scooping with spatula several times to total 25.0 g.

Test portions were extracted and analyzed for OTA using high-performance liquid chromatography with fluorescence detection (Aoyama et al. 2010; Sekiyama et al. 2025). Briefly, test portions were extracted by shaking with acetonitrile-water (3:2, v/v) and then centrifuged. Supernatant was diluted with buffer, filtered through a glass fiber filter, and an aliquot was purified using immunoaffinity chromatography. The methanol-acetic acid (98:2, v/v) eluate was dried down at 45 °C using N_2_ and then reconstituted in water-acetonitrile-acetic acid (70:30:1, v/v) and centrifuged prior to separation and quantitation. Sample extracts were chromatographed using an isocratic program on an octadecylsilylated silica gel column (5 μm particle size, 4.6 × 250 mm) using water-acetic acid (43:2, v/v) and acetonitrile at a ratio of 45:55, (v/v). Excitation and emission wavelengths of 333 nm and 460 nm, respectively, were used to analyze OTA. Concentrations of OTA were not recovery corrected. At the time of collaborative validation, percent recoveries of OTA from wheat fortified at 0.5 µg/kg and 1.5 µg/kg averaged 100% (range 91–110%) and 98% (range 92–102%), respectively, with relative standard deviation under reproducibility conditions 7.7% and 4.4%, respectively. The LOQ was 0.15 µg/kg, determined using a signal-to-noise ratio of 10. Blank wheat and barley matrix samples fortified with OTA at 1.0 µg/kg were run concurrently with analysis to monitor method performance; recoveries obtained ranged from 85 to 120% for wheat and 77–120% for barley.

### Data analysis

Statistical analyses and graphing were performed using R Statistical Software (v4.4.2; R Core Team 2024) with package “ggpubr” (Kassambara 2025). As the distribution of concentrations of OTA does not follow a normal distribution, non-parametric statistical tests were used with the significance level of 0.05.

## Results and discussion

In total, 330 samples from 165 bulk consignments were analysed for OTA at loading and unloading. A summary of the results is provided in Table 1. Since OTA was only observed in one of the barley samples, discussion will focus on common wheat (hereafter referred to as “wheat”) and durum wheat (hereafter referred to as “durum”) samples. Some samples of wheat and durum contained quantifiable concentrations of OTA. However concentrations were low, with all instances of occurrence lower than the Codex Alimentarius Commission Maximum Level of 5 µg/kg (Codex Alimentarius Commission 2023). Quantified concentrations were similar to other reports of OTA in wheat and durum shipments not only from Canada but also from another country (De Pace et al. 2012; Tittlemier et al. 2014; Sekiyama et al. 2025).

**Table 1:** Occurrence of Ochratoxin A (OTA) in paired samples representing 165 bulk consignments. Limits of quantitation (LOQ) were 1.0 µg/kg at loading and 0.15 µg/kg at unloading

	Barley (*n* = 10)		Durum (*n* = 21)		Wheat (*n* = 134)	
	Loading	Unloading	Loading	Unloading	Loading	Unloading
% > LOQ	0	10	19	81	7	51
% ≥ 1.0 µg/kg	0	0	19	14	7	14
Maximum (µg/kg)	-	0.62	2.7	3.3	3.3	3.2

A higher proportion of samples taken at unloading contained quantifiable OTA, due to the lower LOQ of the analytical method used at unloading as compared to loading (Table 1; 0.15 vs. 1.0 µg/kg). When only results that were ≥ 1.0 µg/kg were considered at both loading and unloading, the proportions of samples containing OTA at or higher than 1.0 µg/kg did not differ significantly between samples taken at loading (7%) and unloading (14%) for wheat (Two proportions Z-test; *p* = 0.077). The proportions were not significantly different for durum either (19% vs. 14%; *p* = 0.68). As anticipated, the z-test of all the results of wheat and durum together (total of 155 pairs) also confirmed that there is no significant difference between the samples at loading and unloading. This suggests that OTA was not being produced during the 3 to 5 week period of trans-Pacific and intra-Japanese transport.

There were 32 pairs of loading and unloading samples among wheat (*n* = 25) and durum (*n* = 7) in which the concentrations were mathematically comparable, i.e., at least one member of the pair contained concentrations higher than the highest LOQ of the two analytical methods, 1.0 µg/kg (Table 2). Coefficient of determination (R^2^) calculated using the Spearman’s rank correlation test on these 32 samples each from loading and unloading, assuming < LOQ as LOQ/2, was 0.3. Table 2 indicates that concentrations of OTA were not consistently greater in unloading as compared to loading samples from the same bulk consignment. The Wilcoxon signed-rank test of these results, using the data below the LOQ as LOQ/2, indicated that there was no significant difference between the paired concentrations at loading and those at unloading (*p* = 0.14). This confirms the above conclusion that there was no production of OTA during the trans-Pacific transport as well as when OTA concentrations were examined.

**Table 2:** Ochratoxin A (OTA) ≥ 1.0 µg/kg in either paired samples obtained during loading or unloading bulk consignments

Grain	OTA (µg/kg)		Ratio^a^ (Unloading OTA/Loading OTA)
	Loading	Unloading	
Wheat	1.1	< 0.15	0.07
Wheat	1.1	0.92	0.84
Wheat	1.3	0.20	0.15
Durum	1.3	0.25	0.19
Wheat	1.3	0.36	0.28
Durum	1.3	0.97	0.75
Wheat	1.3	1.1	0.83
Wheat	1.3	2.2	1.7
Durum	1.4	0.24	0.17
Wheat	1.4	1.5	1.1
Wheat	1.8	0.74	0.41
Wheat	2.1	1.7	0.82
Durum	2.7	0.86	0.32
Wheat	3.3	0.74	0.22
Wheat	< 1.0	1.1	2.1
Wheat	< 1.0	1.2	2.4
Wheat	< 1.0	1.3	2.5
Wheat	< 1.0	1.3	2.6
Wheat	< 1.0	1.3	2.5
Wheat	< 1.0	1.4	2.8
Wheat	< 1.0	1.4	2.7
Wheat	< 1.0	1.4	2.7
Durum	< 1.0	1.5	3.0
Wheat	< 1.0	1.6	3.1
Wheat	< 1.0	1.6	3.2
Wheat	< 1.0	1.9	3.8
Durum	< 1.0	2.0	3.9
Wheat	< 1.0	2.3	4.5
Wheat	< 1.0	2.3	4.6
Wheat	< 1.0	2.6	5.1
Wheat	< 1.0	3.2	6.5
Durum	< 1.0	3.3	6.5

^a^ Results < 1.0 or < 0.15 were set to 0.5 or 0.075 µg/kg, respectively, for calculation of the ratio

**Fig. 2:**
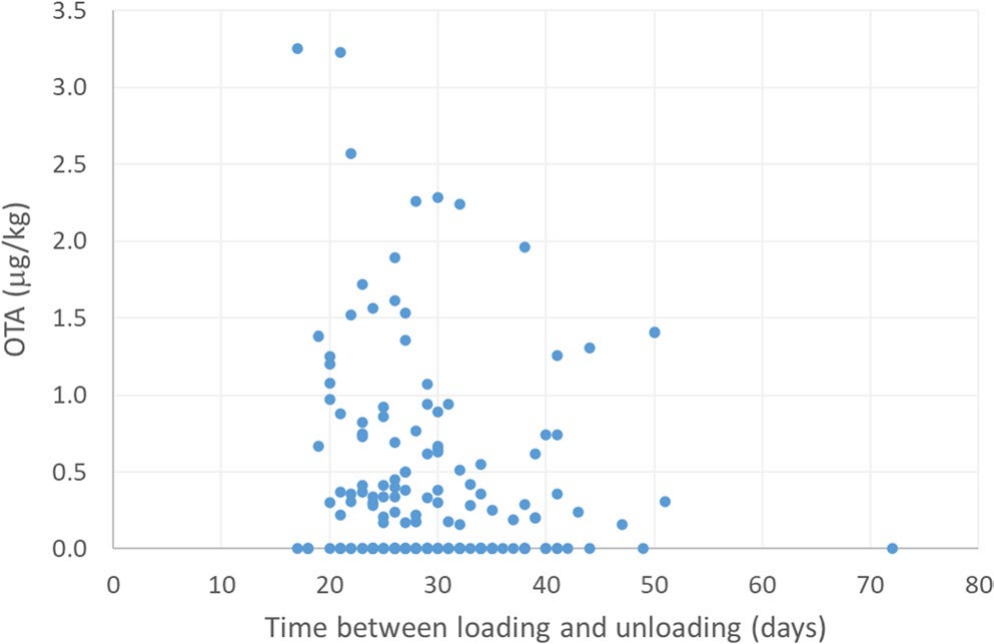
Ochratoxin A (µg/kg) concentrations in unloading samples versus the time elapsed (days) between sampling at loading and unloading. Concentrations less than the limit of quantitation of 0.15 µg/kg were set to 0.075 µg/kg

In addition, there was no correlation between OTA concentration at unloading and the duration of transport in the 165 bulk consignments, including those of barley (Fig. 2), calculated as the time elapsed between sampling at loading and unloading (Spearman’s rank correlation coefficient=−0.147, *p* = 0.058). These results suggest that the conditions in which the bulk grain experienced during transport were not conducive to *P. verrucosum* proliferation and OTA production.

Studies have demonstrated that the *P. verrucosum* population grew with increases in temperature and water activity of stored wheat. Oluwakayode et al. (2024) reported that OTA exceeded 1.0 µg/kg in laboratory storage experiments at 15 and 20 °C after 20 days of storage when water activity was at least 0.9, equivalent to a moisture content of 22%. Similarly, Abramson et al. (2005) reported that OTA exceeded 1.0 µg/kg in wheat stored in a grain bin at approximately 12–17 °C after 28 days of storage when the moisture content was 20%. No OTA was observed in similar samples maintained at 16% moisture content. The mean moisture content for CWRS and CWAD trans-Pacific shipments over the period of this surveillance was 13.4%, ranging from 11.6 to 14.4% and 12.1% ranging from 10.8 to 13.6%, respectively (Canadian Grain Commission, unpublished data). These moisture contents were therefore not expected to facilitate OTA production during transportation to Japan.

Since temperature is intertwined with moisture content as a factor impacting fungal growth and mycotoxin production (Canadian Grain Commission, 2024), results from wheat storage experiments were used to approximate temperature changes that may have occurred in the bulk grain mass during transport. Research by Jayas et al. (1994) demonstrated slow temperature changes in large grain masses. The temperature at the center of approximately 544 t of stored wheat reached its minimum temperature of 10 °C about 150 days after the ambient air reached its minimum temperature of −19 °C. Over a span of 29 days, the mean time between loading and unloading for the 165 shipments with median mass of 26,833 t in this study, there was minimal change in temperature at the geometric center of 544 t of wheat at 25 °C when the ambient temperature increased from 8 to 15 °C. While larger masses of stored grain usually had large temperature gradients and subsequently greater moisture migration and potential for fungal growth and mycotoxin production (Jayas 1995), there was no correlation observed between mean OTA (i.e. mean of results from loading and unloading samples) and mass of the bulk consignments (Spearman’s rank correlation coefficient = 0.006, *p* = 0.94).

Since there was no evidence of OTA concentration changes during transport, the paired results from samples taken at loading and unloading were then considered to be independent samples of the bulk grain mass of each consignment. These data can then be used to characterize the heterogeneity of the bulk grain with respect to OTA. Out of the 32 pairs of wheat and durum samples at least one of which contained OTA higher than 1.0 µg/kg, there were 13 pairs where both the loading and unloading samples had quantifiable concentrations (Table 2). The variability in these paired sample results reflect variance from sampling, sample processing, and analysis, with sampling making the largest contribution (Whitaker et al. 2015).

There are a very limited reports of data that describe the heterogeneous distribution of contaminants such as mycotoxins in bulk commodities, with only two examining OTA in wheat. Ratios of unloading to loading OTA concentrations for the above 32 consignments ranged from 0.07 to 6.5, somewhat lower than the ratios of 5–46 reported for OTA in 500 t increments of bulk wheat consignments (Tittlemier et al. 2025) and a ratio of 43 for OTA in 100 × 100 g increments from a 26 t truckload of wheat (Biselli et al. 2008). It should be kept in mind that study differences, such as experimental design and method LOQs will influence the ratios noted, particularly when aggregate samples representing bulk consignments were analyzed as compared to increments of consignments (Biselli et al. 2008; Tittlemier et al. 2025). Nevertheless, the results from this study demonstrate the uneven distribution of OTA in the bulk consignments due to the biological and agronomic factors influencing OTA occurrence such as the saprophytic nature of *P. verrucosum* and grain handling practices relevant for these large consignments (Tittlemier et al. 2025). Localized proliferation of *P. verrucosum* has also been noted in small areas of stored grain with higher moisture and temperature, leading to pockets of OTA production in storage bins with debris, openings allowing moisture infiltration, or inadequate aeration (Limay-Rios et al. 2017).

Inappropriate sampling methods on heterogenous grain volumes may cause discrepancies between analytical results of individual consignments at loading and unloading, which may lead to trade disputes. Discrepancy of analytical results can also be influenced by the use of different analytical methods; however sampling and sample processing have the greatest impact (Whitaker et al. 2015). In the current study, both analytical methods were objective analytical methods, and the statistical analysis of the overall results indicated no significant difference between the results at loading and unloading, although the method used at unloading employed purification with immunoaffinity chromatography leading to much lower LOQ than the method used at loading.

The main difference in the sampling methods used at loading and unloading was that sampling occurred during the entire loading, and the sample obtained represented 100% of the consignment whereas sampling at unloading occurred at one berth and therefore the sample obtained represented approximately 20% of the consignment. Logistical constraints influenced these sampling differences, since loading took place at one port, which enabled sampling from 100% of the load, and unloading in Japan took place at multiple ports, which made it difficult to sample from 100% of the whole cargo. Additional differences in sampling equipment and processes used (automated cross-cut samplers or manual sampling using a hand scoop; numbers of increments taken from the bulk to form the aggregate) would also impact the results (Andersson et al. 2011). It is not possible to investigate the relative importance of these factors on the comparison of OTA in loading and unloading samples in this study design. However, the results from this study do directly reflect the current reality of international trade, where exporting and importing countries use different sampling methods.

Overall, this work emphasizes the importance of proper sampling and sample preparation to mitigate the impact of the heterogeneous distribution of OTA in large masses of grains such as wheat, including sampling throughout the entire loading process and comminuting large laboratory sample sizes prior to subdivision, on the variance of OTA measurements. While there were no discrepancies between testing at loading and unloading regarding compliance of OTA concentrations with the Codex Maximum Level of 5 µg/kg, adherence to good sampling and preparation practises would help avoid discrepancies and minimize the total measurement uncertainty associated with OTA test results.

## Data Availability

All data supporting the findings of this study are available within the paper.
